# Influence of Coffee Variety and Processing on the Properties of Parchments as Functional Bioadditives for Biobased Poly(*butylene succinate*) Composites

**DOI:** 10.3390/polym15142985

**Published:** 2023-07-08

**Authors:** Mirko Rennert, Benedikt T. Hiller

**Affiliations:** Institute for Circular Economy of Bio:Polymers at Hof University (ibp), Hof University of Applied Sciences, 95028 Hof, Germany; benedikt.hiller.2@hof-university.de

**Keywords:** bioeconomy, coffee by-product, coffee parchment, poly(*butylene succinate*), biopolyester, degradation, stabilization

## Abstract

Fermented polymers like biobased poly(*butylene succinate*) (BioPBS) have become more relevant as technical substitutes for ductile petrochemical-based polymers but require biogenic functional additives to deaccelerate undesired thermo-oxidative degradation and keep a fully biobased character. In this paper, the influence of coffee parchment (PMT) from two different varieties and processings on the thermo-oxidative stabilization and mechanical properties of poly(*butylene succinate*) composites up to 20 wt.-% PMT were investigated. Micronized with a TurboRotor mill, both PMT powders differ in particle size and shape, moisture ab- and adsorption behavior and antioxidative properties. It could be shown that pulped-natural PMT consists partially of coffee cherry residues, which leads to a higher total polyphenol content and water activity. The homogeneous PMT from fully washed processing has a higher thermal degradation resistance but consists of fibers with larger diameters. Compounded with the BioPBS and subsequent injection molded, the fully washed PMT leads to higher stiffness and equal tensile strength but lower toughness compared to the pulped-natural PMT, especially at lower deformation speed. Surprisingly, the fully washed PMT showed a higher stability against thermo-oxidative decomposition despite the lower values in the total phenol content and antioxidative activity. The required antioxidative stabilizers might be extracted at higher temperatures from the PMT fibers, making it a suitable biogenic stabilizer for extrusion processes.

## 1. Introduction

**Figure 1:**
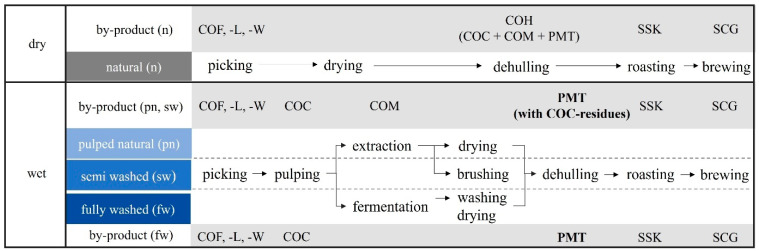
Overview of chronological coffee production from harvest to brewing and the processing-derived by-products in dependence of dry- or wet-processing (COF—coffee flower, COL—coffee leaf, COW—coffee wood, COC—coffee cherry, COM—coffee mucilage, COH—coffee husk, SSK—silver skin, SCG—spent coffee ground & PMT—coffee parchment; during all the processing steps also CD—coffee defects, i.e., immature and defective green coffee beans, are generated).

Coffee is one of the most traded consumer goods in the world and its cultivation has been known for hundreds of years [1]. The technological recycling of its by-products, representing 97 wt.-% of the total mass, is quite new. Along the supply chain from harvesting to brewing, the by-products have a great potential for secondary food, agricultural or material applications [2,3,4,5,6,7]. Most coffee by-products still end up in the environment. Natural decomposition leads to greenhouse gas emissions and their combustion to additional hazards. Residual phytotoxic compounds (e.g., caffeine, chlorogenic acid) can have negative effects on soil and plant growth or on animal health [8,9]. The coffee parchment (PMT) is one of the skins of the green coffee bean and separately generated during wet-processing coffee production. After washing and drying of the extracted (pulped-natural, pn) and fermented (fully washed, fw) coffee bean, the PMT is dehulled from the coffee bean (see Figure 1). The PMT corresponds to a mass fraction of ca. 40 wt.-% of the entire coffee cherry [3]. Brazil alone, the world’s largest coffee producing country, produces approximately 600 kt of PMT annually [10]. The main characteristic of the PMT is its lignocellulosic composition and its high fiber content of approx. 90% [3]. The PMT mainly consists of (α-)cellulose, hemicellulose, and lignin. According to literature, the exact amounts of the lignocellulosic components and the allocation of the polysaccharides partly differ depending on the variety, coffee processing, the solid state of the PMT and the testing.

It is assumed that cellulose forms the main part with approx. 40–50%, compared to 25–35% of hemicellulose and 10–30% of lignin [6,11,12]. Most of these complex macromolecules are insoluble and predestinated as dietary fibers or for material usage. The coffee parchment is supposed to act as semi-permeable barrier layer in the coffee fruit. It has a high water uptake capacity and can act as an oxygen and water vapor scavenger [6]. In dependence of the coffee processing, proper drying is of high relevance to adsorb the free water from the coffee bean and coffee by-products to prevent fungal growth and mycotoxin production. The drying or storage temperatures, moisture content and the water activity a_w_, an indicator for the amount of free water that is available for microorganism and molecular transformation, are the parameters with the greatest influence on the stability of a food or biogenic by-product [13]. For green coffee beans, a water activity a_w_ < 0.80 has shown no fungal growth and should therefore not be exceeded [14].

Today, coffee PMT is simply composted or used for energy recovery after pelletizing the residual biomass. In both processes, CO_2_ is generated. Coffee parchment has high O/C and H/C ratios and thus lower heating values, resulting in a lower efficiency for energy recovery compared to coffee leaves and wood [10]. The PMT also consists of vitamins and phenolic compounds, e.g., caffeine, chlorogenic acid, gallic acid with antioxidative properties. This makes it attractive to the use as additive against oxygen-induced degradation processes [6]. Mirón-Mérida et al. [15] reported that the antioxidative activity of residual PMT from Coffee Arabica variety Costa Rica show antifungal properties in gellan gum films as biobased packaging alternative. Added to bread, PMT can increase the oxidative stability [16]. For a reliable antioxidant effect and a homogeneous distribution of the biogenic additives in the polymer melt, a fine powder with a high specific surface is needed. Slukova et al. [17] and Niemi et al. [18] reported that the TurboTotor milldryer allows high throughputs and fine micronized particles of temperature-sensitive biomass at short processing times. A short and temperature-sensitive drying, milling, and extrusion is necessary to avoid premature decomposition and transformation of the antioxidants of micronized biogenic by-products [19,20] The use of PMT as a stabilizer for biopolymers against thermal, thermo-oxidative or hydrolytic degradation has not been reported yet.

**Figure 2:**
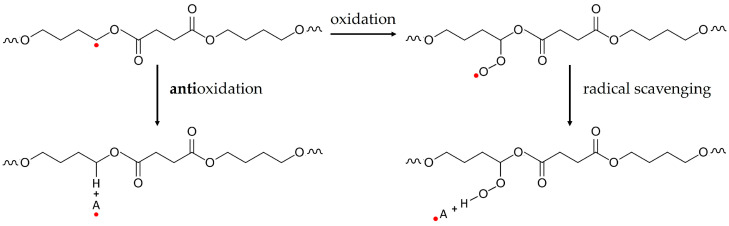
Schematic thermo-oxidative reaction of poly(*butylene succinate*) and (**left**) the mechanism of antioxidant action preventing oxidation or (**right**) eliminating oxidized triggers by radical scavenging (the red points • demonstrate free radicals, A = antioxidant).

Biopolymers can also be made from residual biomass. Fermented biopolymers allow a broader spectrum of initial biomass feedstock with high carbon content and an energy-effective polymerization done by bacteria. Biobased poly(*butylene succinate*) (BioPBS) is an aliphatic biopolyester, made by bacterial fermentation mainly of glucose, e.g., from cellulose resources [21]. It is a semi-crystalline white-appearing thermoplastic polymer with a density of ca. 1.25 g/cm³ and comparable properties to polyethylene. Thus, BioPBS and its copolymers are attractive biobased substitutes to conventional ductile polyolefins. The degree of crystallinity of the PBS homopolymer is higher than for grades with adipate comonomers (PBSA). Under strain, PBS becomes polymorph with additional *β*-crystals [22]. The PBS composition contains an ester group, which is hydrolyzed under water and allows full biodegradation [23]. The understanding and prevention of its degradation during extrusion, i.e., thermal and thermo-oxidative degradation processes, is of high relevance for reliable properties during its application and a multiple recycling in the technosphere. For the thermo-oxidative degradation, Rizarelli et al. [24] showed that the initial decomposing step is the α-hydrogen abstraction from the methyl group next to the ester group (see Figure 2), independently of the macromolecular composition of the PBS/A. The resulting hydroperoxide intermediates lead to a further decrease in molecular weight and the formation of oligomers and macroradicals with different oxidized end groups by radical reaction of the hydroxyl ester or the free radical [24]. Natural antioxidants, i.e., polyphenols bound to the BioPBS macromolecule by hydrogen bonds, reduce free radicals by converting themselves into more stable radicals, interrupting radical chain reactions or eliminating chain reaction triggers by radical scavenging (see Figure 2) [25].

Under exclusion of oxygen, higher temperatures lead to hydrogen abstraction from the *β*-carbon of the succinic acid with the subsequent formation of succinic anhydride, which is declared to be moderately toxic [24]. They also showed that the degradation processes are time dependent. Hallstein et al. [26] were able to confirm thermo-oxidative decomposition due to radical chain reactions. They observed that the PBS molecules start to branch after multiple extrusion cycles and a certain time under the influence of temperature. The addition of phenolic and phosphatic additives neutralized the formation of free radicals and prevented chain branching [26].

The need for stabilization against process- and application-related degradation or changes in the polymer structure makes additives indispensable to obtain a functional bioplastic. A 2020 published study investigated the toxicity of commercially available polymers and biopolymers [27]. Although in most cases more than 1000 different chemicals per sample were extracted under laboratory conditions with solvents and their presumed origin was also addressed, the general conclusion was that biobased and/or biodegradable plastics are at least as toxic as conventional plastics. Less emphasis was placed on the fact that the same conventional additives, classified as toxic and found in conventional plastics, were also found in the bioplastics. Since naturally generated biopolymers can degrade to partially toxic oligomers and phytotoxins that are also often naturally produced by plants, a relativization of the dangerousness of the toxic character of polymers and their ingredients has to be done. Most plant based polyphenols can act as antioxidants with beneficial properties, but also as precursor for toxic compounds [28]. Caffeine is such a toxin, produced by coffee plants to defend themselves. Acting also as an antioxidant, it can have many positive health-promoting and radical scavenging effects [29]. To get fully naturally prestabilized composites based on BioPBS, coffee parchments, containing several antioxidants like caffeine, were added to investigate their influence on the mechanical properties as well as thermal and thermo-oxidative stabilization behavior.

## 2. Materials and Methods

### 2.1. Matrix

The used biobased poly(*butylene succinate*) grade BioPBS^TM^ FZ71PM (PTT MCC Biochem Co., Ltd., Bangkok, Thailand) was made from biobased succinic acid and 1,4-butanediol. The semi-crystalline biopolyester has a density of 1.26 g/cm³, an MFI of 22 g/10 min, a melting point of 115 °C, yield stress of 40 MPa, a stress at break of 30 MPa and a strain at break of 170% (data from the suppliers TDS) and is unprestabilized.

### 2.2. Coffee Parchments

**Figure 3:**
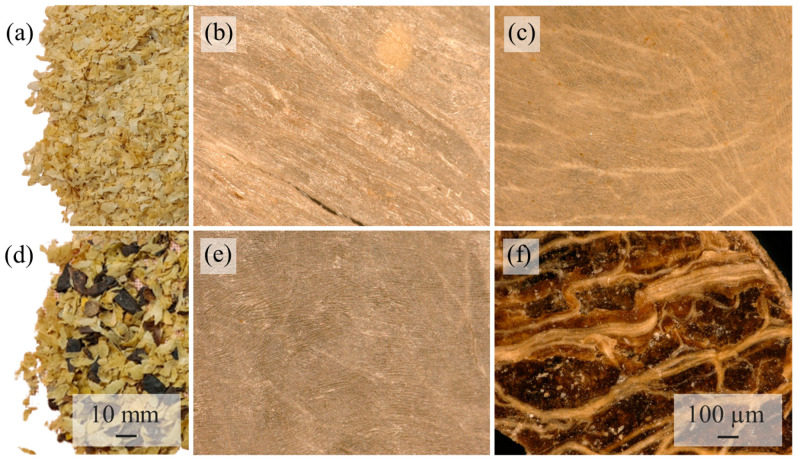
Light microscopic images of the investigated native parchments (**a**) native PMT-SLN fully washed parchment with homogenous PMT; (**b**) native PMT-SLN fully washed parchment in reflective light microscopy with fibrous structure: (**c**) native PMT-SLN fully washed parchment in dark-field light microscopy; (**d**) native PMT-PDD pulped-natural parchment with COC residues (dark brown parts); (**e**) native PMT-PDD pulped-natural parchment in reflective light microscopy; (**f**) native COC-residues-Palha Do Despolpado in dark-field light.

Two coffee parchments were used and kindly provided by Coffee Consulate, Mannheim. The first PMT is from the coffee variety SLN274 (PMT-SLN), a Coffee Canephora, and was picked in 2020 at the plantation Badra Estate (13°21′07.0″ N 75°28′31.8″ E) in India. The coffee was processed fully washed. The second PMT is from C. Arabica, the coffee variety Palha Do Despolpado (PMT-PDD) from the plantation Fazendas Dutra, Brazil (20°18′49.7″ S 42°07′33.9″ W) and was pulped natural processed in 2020. Fully washed (by fermentation) generated PMT are quite homogeneous and almost free of non-PMT parts. Pulped-natural PMT typically have low concentrations of coffee cherry (COC) exocarp or mesocarp because of the extraction-based wet-processing (see darker parts in Figure 3d).

### 2.3. Coffee Parchment Pretreatment and Analysis

The coffee PMTs were stored in barrier bags during transportation and storage. The fine milling of the PMT was done with a TurboRotor^®^ milldryer from Mahltechnik Görgens GmbH (Dormagen, Germany). The micronization principle is based on a temperature-regulated high air throughput and appropriate for temperature-sensitive materials. The grinding principle is based on turbulences created by the air stream, leading to multiple impacts between the particles and the machine plate. For the PMT, a rotor speed of 113 rpm with a material throughput of 1600 kg/h was chosen. The temperature of the intake air was 136 °C and the exit temperature was 99 °C. The bulk density of the PMT-SLN was 0.255 g/cm³ (native) and 0.430 g/cm³ (micronized), and the bulk density of the PMT-PDD was 0.245 g/cm³ (native) and 0.440 g/cm³ (micronized).

The analysis of the particle size and particle size distribution was done with a CILAS particle size analyzer 1090 (Cilas S.A., Orleans, France), a laser diffraction granulometer at ambient temperature. The PMT powder was diluted 10 times with water and 5 vol.-% tenside to avoid reagglomeration and obtain a homogenous emulsion. Ultrasound (US) was applied to break the aggregates into single particles. The measurement of the intensity-time fluctuations of the particles is done by two laser beams at 635 and 830 nm with 3 × 10 runs. Additionally, the powders were sieved with an Alpine 200 LS air jet sieve (Hosokawa Alpine AG, Augsburg, Germany) of mesh sizes 100 µm, 63 µm, 45 µm and 32 µm and the mass of the fractions was subsequently measured.

The hygroscopic properties of the micronized PMT powder were investigated with a climate chamber and a drying oven. For the humidity absorption, beakers with 150 mL of each PMT powder were stored for 48 h at 85% r.H. and 23 °C. The initial mass of the untreated sample was determined prior to the conditioning. The initial moisture content was measured with the moisture analyzer Kern DBS (Kern and Sohn GmbH, Balingen-Frommern, Germany). The periodic changes in mass were determined to calculate the corresponding moisture contents with respect to the initial moisture content. After 48 h, the samples were transferred to a drying oven at 75 °C for another 48 h with periodical mass loss evaluation. The water activity *a_w_* was measured with the dew point water activity meter WaterLab (Steroglass S.r.l., Perugia, Italy). The *a_w_* gives an indication for the relation of free and bonded water in materials and thus for the stability against biodegradation and hydrolytic reactions during polymer processing.

Thermogravimetric analysis (TGA) was performed with the TG 209 F3 Tarsus^®^ from Netzsch (NETZSCH-Gerätebau GmbH, Selb, Germany) to indicate critical degradation temperatures and the composition of the lignocellulosic structure of the prepared PMT according to the specific mass loss [12]. Each sample was measured in pure N_2_ and an 80/20 N_2_/O_2_ atmosphere in a temperature range of 40–810 °C with a heating rate of 10 °C/min. The micronized PMT samples of 10 mg were analyzed in 85 µL open Al_2_O_3_ crucibles. A sample mass of 9.5 ± 0.5 mg of each powder sample was measured in a temperature range from 40 °C to 550 °C at a constant heating rate of 10 °C/min.

An optical observation of the degradation behavior of the PMT powders was done with a Kofler Heizbank^®^ (Wagner & Munz GmbH, München, Germany) with heating zones from 50–260 °C. In heated state, a 1 mm thick layer of each PMT powder was placed across the metal surface and the color change was recorded after 5 min.

The total phenolic content (TPC) was determined with FC-Assays according to Singleton et al. [30] and the radical scavenging properties were determined with DPPH assays according to Brand-Williams et al. [31] and ABTS assays according to the procedure of Re et al. [32]. A detailed description of the used and modified Assay procedure can be seen at Hiller et al. [33]. For the extraction, 1 g of each PMT was diluted in glass test tubes with 10 mL of 50/50 water/bioethanol and stored for 60 min in a US bath (Allpax GmbH and Co. KG, Papenburg, Germany) preheated at 40 °C. The temperature of the US bath increased to 52 °C after 30 min and 56 °C after 60 min. The liquid extract was refilled into plastic test tubes and placed in a centrifuge (Heraeus Noblelight GmbH, Hanau, Germany) at 3500 rpm for 30 min to separate the liquid and solid fractions. For the FC assay, a Folin–Ciocâlteu reagent was used and calibrated with gallic acid. A volume of 100 µL of the diluted PMT extract (10% *v*/*v*) was added to 7.9 mL of distilled water and 500 µL of FC reagent. After an incubation time of 8 min, 1.5 mL of a sodium carbonate solution (20% *w*/*v* sodium carbonate and distilled water) were added and the solution was incubated again for 30 min in a closed water bath at 40 °C. The absorbance measurements were performed with a DR 6000 UV/VIS spectrophotometer from Hach at 765 nm. The TPC was obtained from the gallic acid calibration curve (R² = 0.997) and then expressed as mg of gallic acid equivalents (GAE) per g dry PMT powder. For the determination of the radical scavenging activity, a 2,2′-azino-bis-3-ethylbenzthiazoline-6-sulphonic acid (ABTS) as radical reagent was used and calibrated with Trolox. The ABTS working solution was prepared with 10 mL of an ABTS stock solution (96 mg ABTS salt in 25 mL distilled water) and 175 μL of a potassium persulfate solution (189.2 mg of potassium persulfate with 5 mL of distilled water) and incubated for 12 h in the dark at ambient lab conditions. The ABTS working solution was subsequently diluted with bioethanol until it reached an absorbance value of 0.700 ± 0.005 at 734 nm in the spectrophotometer. The Trolox standard was diluted with bioethanol to a concentration of 0.5 g/L. For the absorbance measurements at 734 nm, 100 µL of the diluted PMT extract (initial dilution of 20% up to 0.625% *v*/*v*) were added to 3.4 mL of the diluted ABTS working solution. For the DPPH assay, 1,1-diphenyl-2-picrylhydrazyl (DPPH) radical was used and calibrated with Trolox. As with the ABTS assay, this method measures the antioxidant activity in Trolox Equivalent Antioxidant Capacity (TEAC, mg/mL). A 60 μM DPPH/bioethanol solution was prepared and the same Trolox solution was used as for the ABTS. Before the absorbance measurements at 515 nm, 100 µL of PMT antioxidants were added to 3.9 mL DPPH radical solution and incubated for 30 min in the dark at ambient lab condition.

### 2.4. Composite Processing

**Figure 4:**
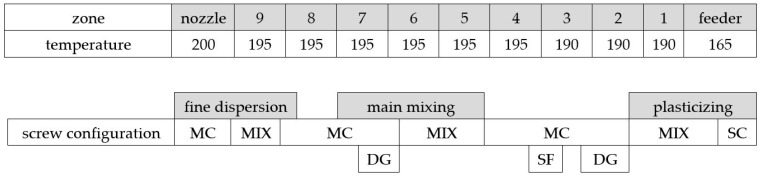
Temperature profile and screw configuration of the twinscrew compounder for the compounding of BioPBS FZ71 and PMT (SC—solid conveying, MIX—kneading & mixing elements, MC—melt conveying, SF—side feeder, DG—degassing).

For the compounding of the BioPBS with the PMT powder a co-rotating twinscrew lab extruder (Labtech Engineering Co., Ltd., Samutprakarn, Thailand) with an L/D ratio of 44 was used. The BioPBS was pre-dried at 80 °C for 5 h in a LUXOR 50 dry air predryer (Motan Holding GmbH, Konstanz, Germany). The PMT was pre-dried at 60 °C for 12 h with a residual moisture of 1.31% (PMT-SLN) and 1.85% (PMT-PDD). The extruder temperature profile and screw configuration can be seen in Figure 4. The screw speed was set at 230 rpm and the throughput at 12 kg/h. The melt temperature at the die varied between 176 and 181 °C.

The PMTs were dosed gravimetrically (Scholz Dosiertechnik GmbH, Großostheim, Germany) in 5, 10, 15 and 20 wt.-% via side feeding. The extruded filament was cooled in a water bath and finally pelletized with a Labtech type LZ-120/hp granulator and dried again at 60 °C for 6 h. Injection molded specimens for tensile (type 4) and Charpy-test (type 1A) were produced with a BOY XS injection moulding machine (BOY Machines, Inc., Exton, PA, USA). The temperature profile from the feeder to the die was 25–155–160–170 °C, using an injection speed of 3 cm³/s, a mold temperature of 25 °C, a holding time of 2 s, a holding pressure of 600 bar max. and a cooling time of 20 s. The process conditions for compounding were selected in terms of melt viscosity, incorporation of PMT up to 20 wt.-%, throughput, cooling and pelletizing, and for injection molding in terms of granule feeding, melt viscosity and filling of mold cavities without overhang.

### 2.5. Composite Analysis

To characterize the mechanical properties the standard tensile test and the Charpy impact test was applied. Tensile testing was performed on a zwickiLine Z2.5 (ZwickRoell GmbH and Co. KG, Ulm, Germany) according to DIN-EN ISO 527-3 to evaluate the tensile modulus *E_t_*, the tensile strength *σ_m_* and the elongation at break *ε_b_*. The gauge length at the starting position was 30 mm and the preload 0.25 N. According to ISO 527, the Young’s modulus for a specimen was determined at a testing speed of 1 mm/min as a secant modulus in a first step up to 0.25% elongation with a subsequent measurement of the tensile properties by switching to a testing speed of 50 mm/min. Since the standard deviation of the tensile specimen for each series was very small, five tensile specimen per recipe were used. Charpy impact tests were done according to DIN EN ISO 179-1 on a Zwick/Roell HIT25P (ZwickRoell GmbH and Co. KG, Ulm, Germany) with a 1 Joule pendulum at ambient lab conditions. The Type 1 injection moulded specimen were single edge notched with a depth of 2 mm by notching machine MAK (Emmeram Karg Industrietechnik, Krailing, Germany). Ten Charpy samples were used for each series.

The Melt Flow Index (MFI) was measured with the MeltFloW basic (Emmeram Karg Industrietechnik, Krailing, Germany) according to DIN EN ISO 1133. The BioPBS was preheated for 300 s at 180 °C and loaded with 2.16 kg.

Gel permeations chromatography (GPC) was done with an Agilent SEC 1100 (Agilent Technologies, Inc., Santa Clara, CA, USA) in duplicate. The eluent of the system was hexafluoroisopropanol (HFIP) with the addition of 50 mM sodium trifluoroacetate at 30 °C. A flow rate of 1 mL/min was used. Calibration was performed with polymethyl methacrylate standards. The injected sample volume was 100 μL.

Light microscopic images were recorded with a digital microscope VHX 950F (KEYENCE DEUTSCHLAND GmbH, Neu-Isenburg, Germany) in reflective and dark-field mode. For the scanning electron microscopy (SEM), the samples were sputtered with a Cressington Carbon Coater 108 (TESCAN GmbH, Dortmund Germany) and measured with a Jeol 6360 (Joel Ltd., Tokyo, Japan).

The investigations of the thermal properties were done with TGA and differential scanning calorimetry (DSC). For TGA a TG 209 F3 Tarsus (NETZSCH-Gerätebau GmbH, Selb, Germany) and the same parameters were used as for the native PMT. The DSC measurements were performed with a DSC 214 Polyma (NETZSCH-Gerätebau GmbH, Selb, Germany). Standard DSC was performed with two heating and cooling runs between −60–180 °C at a heating rate of 20 °C/min. Glass transition temperature ($T_{g}$), melting temperature ($T_{m}$) and the melting enthalpy ($\Delta H_{m}$) were obtained from the second heating run and the crystallization enthalpy ($\Delta H_{c}$) were obtained from the cooling cycle. The degree of crystallinity (${Χ}_{C}$) was calculated using the Equation (1)

(1) $${Χ}_{C}\;\%=\frac{\Delta H_{c}}{\Delta H_{m}^{0}\left(1-w\right)}\times 100$$

with the mass fraction (w) of the PMT and the theoretical melting enthalpy of 100% crystalline BioPBS ($\Delta H_{m}^{0}$) according to Miyata et al. [34]. Dynamic Oxidation Induction Temperature (OIT) analysis was performed according to DIN EN ISO 11357-6 with the onset and offset method.

## 3. Results and Discussion

### 3.1. Coffee Parchments

**Figure 5:**
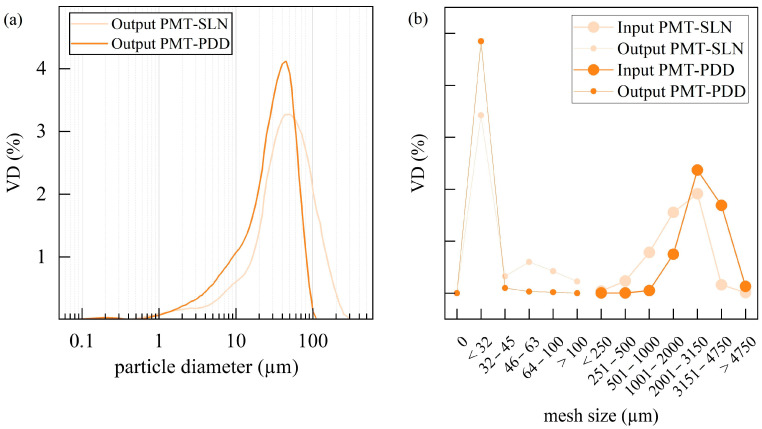
Particle size and particle size distribution of the two fine grinded coffee parchment varieties PMT-SLN274 and PMT-Palha Do Despolpado, analyzed with the laser diffraction granulometry (**a**) after the grinding and the air jet sieving (**b**) before (=input) and after the grinding (=output).

Particle sizes and particle size distributions from laser diffraction and air jet sieving can be seen in Figure 5. In particular, the particle sizes of the native PMT by-products depend on the dehulling process. The smaller the initial size of the by-product, the smaller the fine milled powder using identical grinding parameters usually is. The particle sizes of the native PMT-PDD shifted towards larger values compared to PMT-SLN, mainly due to bigger COC residues. This can also be seen in the native/final bulk density of 0.255/0.43 g/cm³ for the PMT-SLN and 0.245/0.44 g/cm³ for the PMT-PDD. Interestingly, after fine milling with the same process parameters, the PMT-PDD have an overall lower particle size with the granulometry-measured top cut D_97_ of 93.5 µm, compared to a D_97_ of 168.2 µm for the PMT-SLN. The particle size distributions are comparable for both varieties. A similar trend can be seen for the results of the air jet sieved powders, but 97% of the PMT-PDD particles are smaller than 32 µm, compared to the 68.5% for the PMT-SLN. Even if a surfactant and US were used in the wet-based granulometry, reagglomeration, especially of particularly small particles, can occur during the measurement due to friction. For higher particle sizes, both methods reveal no values greater than 100 µm for the PMT-PDD, but a considerable low number of large particles for the PMT-SLN.

**Figure 6:**
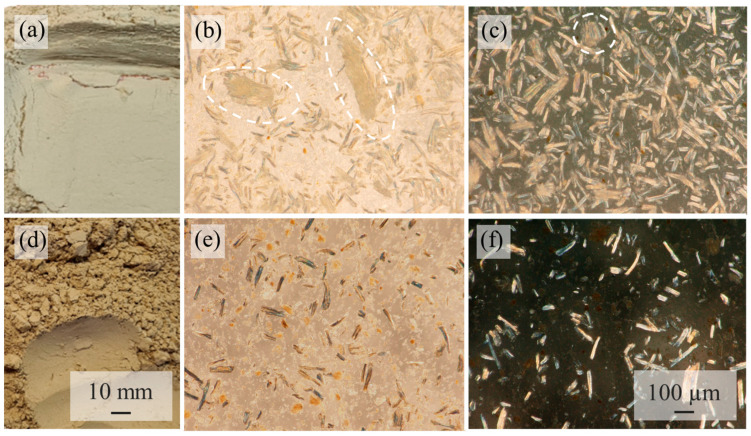
Microscopic images of the milldried PMT by-products (**a**) PMT-SLN powder; (**b**) PMT-SLN in reflective light microscopy; (**c**) PMT-SLN in dark-field light microscopy; (**d**) PMT-PDD; (**e**) PMT-PDD in reflective light microscopy; (**f**) PMT-PDD in dark-field light.

A possible explanation for this is shown by the microscopic images of the powders in Figure 6. The PMT powder of the SLN shows a higher concentration of fibers than the PMT-PDD. Irionda-DeHond et al. [35] found total dietary fiber contents >92% for C. arabica PMT with almost no soluble fibers and only 72% total fibers for COC with a soluble fraction of 12%. The PMT-SLN has no COC fractions and thus a higher total fiber content compared to the pulped natural processed PMT-PDD with COC residues. The separation of PMT from the COC of the PMT-PDD resulted in a mass fraction of 61.22 wt.-% PMT and 38.78 wt.-% COC. Regarding the location and the height of coffee cultivation, it could be shown that the conilon coffee cherries of the C. canephora variety EMCAPER 8151 from Brazil have a higher total fiber content, if they are grown in southeast-facing slopes compared to northwest-facing slopes. The influence of the altitude is even bigger. Higher altitudes are associated with lower ambient temperatures and thus a slower ripening and building of functional compounds and higher molecular structures [36].

**Figure 7:**
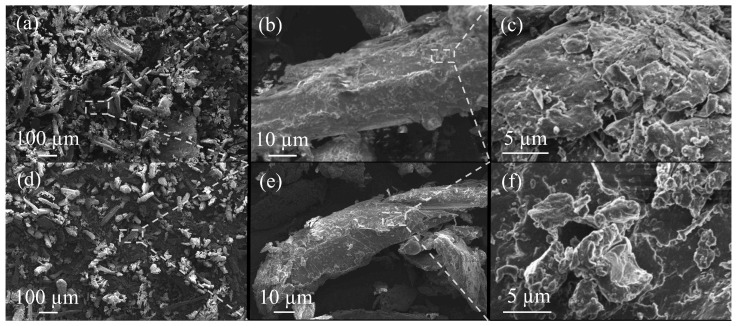
SEM images of the parchment by-product grinded powder of the PMT-SLN (**a**–**c**) and PMT-PDD (**d**–**f**).

Fibers of coffee by-products are classified as fine and short. The fiber geometry of the PMT-PDD is more homogenous with smaller fiber diameters (Figure 6 and Figure 7). The fibers of the PMT-SLN are different as seen in Figure 6b,c. The average fiber length of the PMT-SLN is 187.1 ± 45.3 µm with an average fiber diameter of 35.5 ± 25.8 µm, compared to a smaller fiber length of 87.1 ± 49.3 µm with a smaller fiber diameter of 24.2 ± 12.3 for the PMT-PDD. Bekalo et al. [11] investigated coffee parchment fibers with lengths between 50 and 800 µm and diameters around 15 µm, which are in accordance to the average sizes for the PMT-SLN and PMT-PDD. Benitez et al. [37] reported comparable quantitative PMT compositions independent of the coffee processing, but potential quantitative variations in cellulose, hemicellulose and lignin contents, which might lead to different fiber geometries. The micronization process is supposed to lower the lignin content significantly [37]. Since both PMT were milldried using identical process parameters, PMT-SLN seems to have stronger and more heterogeneous fibers.

**Figure 8:**
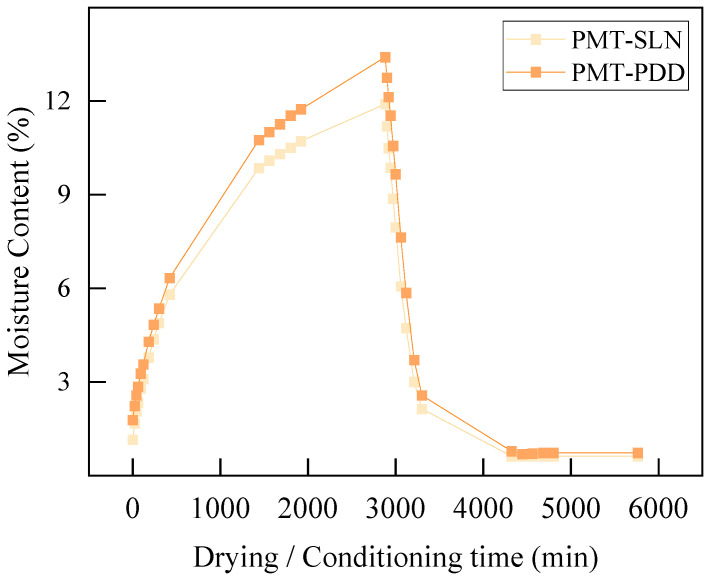
Water absorption (48 h at 23 °C and 85% r.H.) and adsorption (48 h at 75 °C) curves for the two fine grinded coffee parchment varieties PMT-SLN274 and PMT-Palha Do Despolpado.

To avoid biological degradation and molecular transformations during storage, the PMT powders need to be dry and protected from the influence of oxygen, moisture and UV radiation. A permanent moisture content < 13% is required to avoid biological degradation and the formation of mycotoxins due to fungal growth [38]. The initial moisture contents were 7.0% for the native PMT-SLN and 10.3% for PMT-PDD. Thus, the relative moisture of both by-products was below the critical limit for mold growth (see Table 1). The higher equilibrium moisture content of the PMT-PDD might be related to the COC fraction and the associated sugar content due to the pulped natural process. During the extrusion, residual moisture can lead to hydrolytic degradation and air cavities in the melt. Figure 8 shows the results of the humidity absorption and adsorption test of both PMT. The milldried PMT-SLN had a lower initial moisture content of 1.16% compared to 1.79% for the PMT-PDD, which was already doubled after 60 min. PMT-PDD showed a stronger increase of the moisture content and reached 12% after approx. 30 h, whereas the PMT-SLN did not even reach this moisture content after 48 h.

It may be concluded that the lower particle sizes and corresponding higher specific surface of the PMT-PDD might be the reason for a faster and higher absorption of humidity. The adsorption speed was comparable for both PMT. After 48 h of drying, the PMT-SLN had a residual moisture content of 0.63%. The moisture content of the PMT-PDD was slightly higher with 0.73%. Drying of grinded powders before extrusion is essential and degassing should also always be considered during compounding and extrusion. The results of the water activity measurement *a_w_* are displayed in Table 1. The higher moisture content of the PMT-PDD correlates with the higher water activity *a_w_* of 0.47 for the native parchment due to the COC fractions.

The *a_w_* of PMT-SLN is 32% lower in native state/form and 15% lower after the milldrying. Both parchments show water activities strongly lower than the critical a_w_ values, excluding fungal growth. The residual moisture and particle size of the PMT powders are also relevant for the evaluation of the antioxidative properties, next to the solvents and the duration of the chosen extraction method [39].

Table 1 also shows the results of the evaluation of the Total Phenolic Content (TPC) and Antioxidant Activity (AA). With 8.46 mg GAE/g dw PMT-PDD and 7.47 mg GAE/g dw PMT-SLN, both parchments have an amount of TPC two to four times higher TPC than was found by Benitez et al. [37] and Aguilera et al. [40] from wet-processed C. Arabica parchments by only using water as a solvent and Mirón-Merida et al. [15], who worked with PMT from C. Arabica variety Costa Rica from Mexico. Irionda-DeHond [35] reported a TPC of ca. 68 mg GAE/g PMT dw, a wet-processed var. Arabica from Finca Morenitas from Nicaragua. The higher TPC of the PMT-PDD might be explained by the COC fractions. It was shown that COC and COH (from dry processing with COC) usually have higher TPC values, varying between 9–22 mg GAE/g dw COC [41] up to even 489.5–1810 mg GAE/g dw COP for red cherry pulps from C. Arabica varieties 741, Dessu, 74,110 and Ababuna (hybrid of 741 × Dessu) from Ethiopia. Both, cultivation conditions (location, height, soil composition, climate, etc.) and the extraction method (extract solution, extraction time and treatment, etc.) may influence the TPC and just allow quantitative comparison of similarly treated samples. Nevertheless, the above-mentioned studies have shown the trend that the extracts of the coffee exocarp (COP, COC and COH) resulted in higher TPC than extracts of the PMT. It might be assumed that the anthocyanins, the natural mainly red polyphenolic colorants, which are supposed to stabilize the coffee fruit, are responsible for the higher TPC. After the depulping step during wet-processed coffee production, some mucilage residues adhere to the parchments that might get hydrolyzed, forming various polyphenols [42], which might explain the comparatively high TPC of the wet-processed PMT-SLN. Comparatively high TPC might also be a proof that the gentle milldrying process used in this study prevents degradation of polyphenols during the micronization. The higher sea level of both PMT cultivation sites might also have a positive influence on the TPC as shown by Pereira et al. [36].

The radical scavenging properties were evaluated by DPPH and ABTS assays. The antioxidant activity (AA) can be characterized by potentially hydrogen-donating antioxidants that neutralize the DPPH radicals or chain-breaking antioxidants for ABTS radical neutralization. The PMT-PDD showed a higher AA than the PMT-SLN. With 2.75 mmol TE/100 dw, the AA_DPPH_ of the PMT-PDD with COC-residues is four times higher than the one for PMT-SLN. The main function of the exocarp of the coffee fruit is to protect the coffee bean from oxidative degradation, e.g., by UV radiation. Thus, the majority of antioxidants is expected to be contained in the COP and COC. Vijayalaxmi et al. [39] showed that Tannins seem to be the dominant polyphenols in coffee husks COH, which are rich in COC. This assumption fits well to the results of Cangussu et al. [41], who found AA_ABTS_ values of 7.56 mmol TE/100 g dw for a COH with a COC fraction of approx. 80 wt.-% pulp and peel and 20 wt.-% PMT. Benítez et al. [43] recently assumed that the lower AA of PMT can be ascribed to bound polyphenols within the PMT fibers.

**Figure 9:**
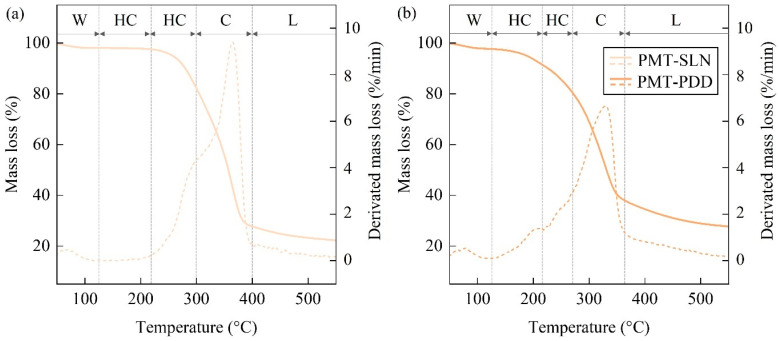
TG- and DTG-curves with the mass loss fraction zones according to [12] for the constituents of the parchment varieties (**a**) PMT-SLN and (**b**) PMT-PDD (W = water; HC = hemicellulose; C = cellulose; L = lignin).

Figure 9 shows the TGA mass loss curves of the PMT. The decomposition rate of the thermogravimetric analysis (DTG) is usually represented by the first derivative of the TGA curve. The process of thermal decomposition of the coffee parchment evolves in five main steps. The first loss of mass corresponds to the evaporation of the remaining free water. The resulting residual moisture contents of 1.6% for PMT-PDD and 1.0% for PMT-SLN are in good agreement with the values of the residual moisture determination after milldrying, where the samples were heated by halogen lamps at 105 °C. The slightly higher moisture content of the initial moisture testing might be a result of additional evaporated volatile organic compounds (VOC). In the temperature range of up to approx. 220 °C, lower molecular weight components of the hemicellulose with lower thermal stability degrade subsequently. Here, a first clear difference between both PMTs can be seen. The PMT-SLN shows almost no further degradation. On the other hand, the PMT-PDD shows a mass loss of up to 6.7 wt.-%. This might indicate a fraction of the less thermally stable hemicellulose. Further, it might be supposed that the COC residues of the pulped-natural processed PMT-PDD contain residual sugars that lead to reduced sugar molecules during thermal treatment. It was shown by Cao et al. [44] and Gancarz et al. [45] that the Maillard reaction, starting during coffee roasting at approx. 140 °C, can create VOC with an associated non-enzymatic browning of the biomass. This assumption is consistent with the studies on the influence of temperature on the discoloration of PMT.

**Figure 10:**
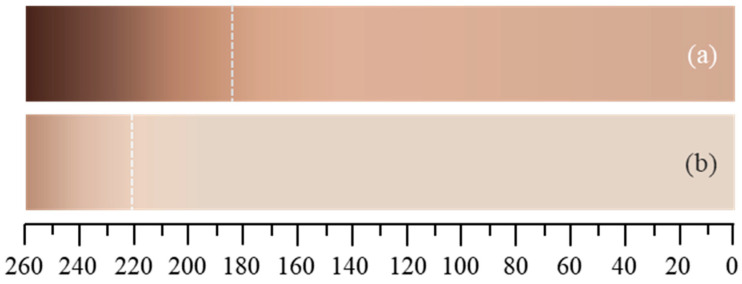
Kofler-Heizbank^®^ with increasing temperature and starting point of thermal degradation in terms of browning for milldried coffee parchment varieties (**a**) PMT-PDD and (**b**) PMT-SLN.

Figure 10 shows the onset of brown discoloration in PMT as a function of temperature. For PMT-PDD, this process starts at significantly lower temperatures (approx. 180 °C) compared to PMT-SLN, which starts to discolor at approx. 220 °C. From this temperature up to 300 °C the PMT-SLN shows a first DTG peak which may be associated with the degradation of the containing hemicellulose.

Benitez et al. [37] reported that the majority of PMT hemicellulose might be Xylans, which are not directly connected to the cellulose. In total, both varieties of PMT have comparable hemicellulosic fractions with ca. 16 wt.-% for the PMT-SLN and 17 wt.-% for the PMT-PDD with respect to the characteristic temperature ranges for thermal decomposition. The main degradation step starts from approx. 300 °C on and is associated with the decomposition of cellulose. The comparatively long linear glucose macromolecules form a thermally stable crystal morphology. The cellulosic DTG for the PMT-SLN is higher in mass loss and at higher temperatures with 364.4 °C compared to a DTG of 329.6 °C for the PMT-PDD. The resulting cellulosic content is 26% higher for the PMT-SLN. The final degradation steps are mainly associated with the lignin content and residual ash contents. Interestingly, the PMT-PDD shows a higher content of complex crosslinked lignin macromolecules, which might have a positive influence on the thermal stabilization of the composites. Table 2 summarizes the TGA results of the parchments powders that reveal a generally higher thermally stability for PMT-SLN.

### 3.2. Composites Based on BioPBS and PMT

The thermal properties of the composites were analyzed by standard DSC and TGA (Table 3). Glass transition temperature ($T_{g}$) and melting temperature ($T_{m}$) are within the expected ranges and in accordance to the suppliers technical data sheets. Type and concentration of by-product have almost no influence on the temperature transition ranges of $T_{g}$ and $T_{m}$. The degree of crystallinity of the BioPBS matrix is in the range of 35% and stays unaffected by the fillers. A nucleating effect, as shown for polyhydroxy-alcanoates (PHA) by lignocellulosic additives, was not observed [46]. For the same PHA, an increased stability against thermal degradation was achieved by the incorporation of coffee PMT due to the higher thermal stability of the lignocellulosic PMT fibers [46]. Such a stabilization effect was not observed for the investigated BioPBS composites. Both PMT show mass losses at slightly lower temperatures with increasing filler content. However, while the DTG of the PMT-SLN composites remains almost constant up to filler contents of 20 wt.-%, the PMT-PDD has a slightly negative impact on the thermal stability, represented by Ton and the DTG peak. This is in accordance with the TGA results of the native PMT, where a higher thermal stability of the PMT-SLN was observed.

**Figure 11:**
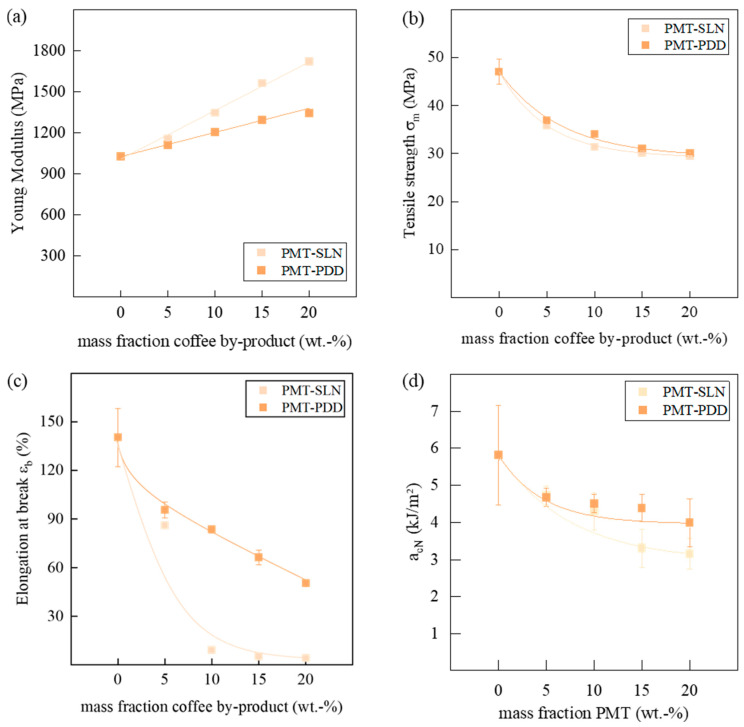
Influence of the variety of coffee parchment and its mass fraction on the (**a**) Young Modulus, (**b**) the tensile strength $\sigma_{m}$, (**c**) the elongation at break $\varepsilon_{b}$ and (**d**) the $a_{cN}$ of the BioPBS compounds.

One promising benefit of parchment fibers is seen in the positive influence on the mechanical properties. As shown in Figure 11a, a linear increase of the tensile Young’s modulus with increasing mass fraction of the PMT can be seen. While the modulus of the PMT-SLN composites with 20 wt.-% is increased by approx. 70%, the modulus of the PMT-PDD increased only about approx. 30%. Reis et al. [47] also found increasing moduli for PHA with increasing PMT fraction. It might be assumed that the filler distribution reduces chain mobility under tensile deformation, as was also shown by Gowman et al. [48] for BioPBS composites. Fractions with higher particle sizes for the PMT-SLN might be the reason for the stronger resistance to deformation. The tensile strength of all PMT composites decreased exponentially in the same dimensions of up to 40% for mass fractions of 20 wt.-% PMT (Figure 11b).

Since the elongation at break also shows a decrease with the addition of the parchment, it is suspected that there are poor matrix-particle bonds (Figure 11c). Weak interfacial interactions can cause cracks, leading to premature fracture. This is particularly evident for the PMT-SLN composites. There, the elongation at break decreases with the addition of 10 wt.-% PMT-SLN from about 150% to 10% and then remains at this level.

**Figure 12:**
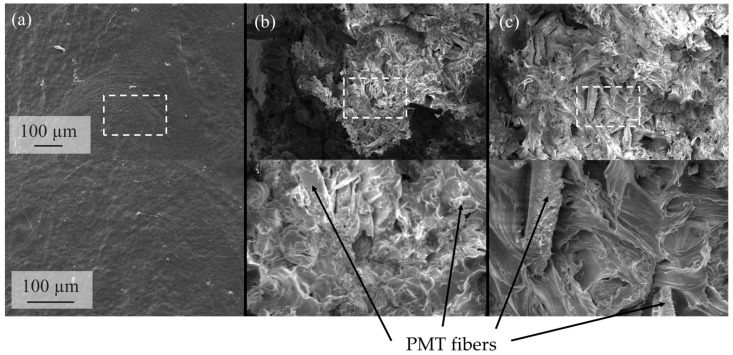
SEM images of the composite fracture surfaces after tensile deformation for the (**a**) neat BioPBS, (**b**) with 20 wt.-% PMT-PDD and (**c**) with 20 wt.-% PMT-SLN.

Since particularly large fiber particles with a homogeneous smooth surface were found in the PMT-SLN, it is reasonable to assume that the defects occur particularly at their interfaces, leading to a reduced ductility and macrocracks. After an initial sharp drop in elongation at break, the composites with PMT-PDD show a linear decrease, still reaching 60% at 20 wt.-% PMT-PDD of the initial elongation at break of neat BioPBS. Figure 12 shows the fracture surface of the neat BioPBS and the composites with 20 wt.-% PMT after tensile deformation. Neat BioPBS specimen showed a ductile deformation bahvior with a necking of the tensile specimen. After crack initiazion an instable crack propagatrion lead to smooth and flat fracture surface (see Figure 11a). For the BioPBS composites at 20 wt.-%, different fracture surfaces were observed between the two varieties. The fracture surface of the BioPBS composite with PMT-PDD shows better adhesive interaction of the particles with the matrix, but also some voids. The generation of many small microsurfaces could indicate stable crack growth and thus higher crack resistance. The fracture surfaces of the PMT-SLN reinforced BioPBS indicate less pronounced matrix-particle adhesion and fiber pull-out. The cavities around the PMT fibers could act as predetermined fracture sites and promote faster crack growth. The BioPBS matrix, while appearing somewhat fibrous, tends to indicate more unstable crack growth, which is consistent with the lower elongation at break at higher PMT concentrations. Reis et al. [47] also observed PMT fiber pull-outs from the investigated PHA matrix and also concluded weak particle-matrix interactions. Consequently, natural compatibilizers and a surface treatment of the PMT fibers seems to be required. A different relationship is seen for mechanical stress applied at higher speeds, where the Charpy impact strength decreases with increasing filler contents. Figure 11d shows the $a_{cN}$ in dependence of the PMT mass fraction. However, this decrease is much less pronounced for both varieties of PMT and is comparable up to 10 wt.-% with slightly lower $a_{cN}$ for higher mass fractions of PMT-SLN. The assumption of an increase in embrittlement can be seen in the increasing standard deviations. Insufficient particle wetting with the BioPBS matrix can lead to accelerated microcrack agglomeration that finally leads to a fracture. The higher standard deviation of the neat BioPBS might be traced back to heterogenous crack propagation and may be associated with crystal morphology. It might be assumed that cracks propagate across the crystal phases and thus have a more uncontrolled fracture behavior. Up to mass fractions of 10 wt.-%, the crack propagation was more homogenous, which might be ascribed to predetermined breaking points.

**Figure 13:**
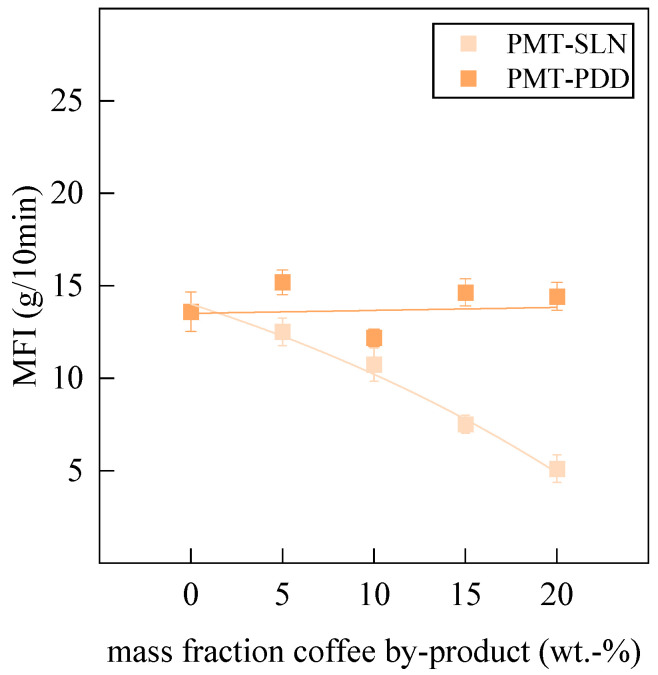
Influence of the variety of coffee parchment and its mass fraction on the MFI of the Bio-BioPBS compound.

Figure 13 shows the rheological results of the MFI testing. The MFI of the PMT-SLN composite shows a linear decrease with an increasing PMT mass fraction. This result is to be expected since the incorporated particles hinder the mobility of the polymer chains to slide off and accordingly increase the viscosity. This is in accordance with the lowered elongation at break, where the energy of mechanical deformations cannot dissipate due to a limited chain mobility. Consequently, higher energy is required to allow full plasticizing of, for example, injected molded products. The specimens, injected molded for this investigation, could be processed with the same extrusion parameters. Interestingly, an opposite result is obtained for PMT-PDD composites. The MFI remains unchanged, regardless of the filler concentration. TGA and the Kofler-Heizbank^®^ indicated reduced sugars caused by the Maillard reaction. Since polycondensation reactions might occur, the subsequent production of water and reduced sugars could act as short-time plasticizer and reduce the viscosity of the polymer matrix.

**Figure 14:**
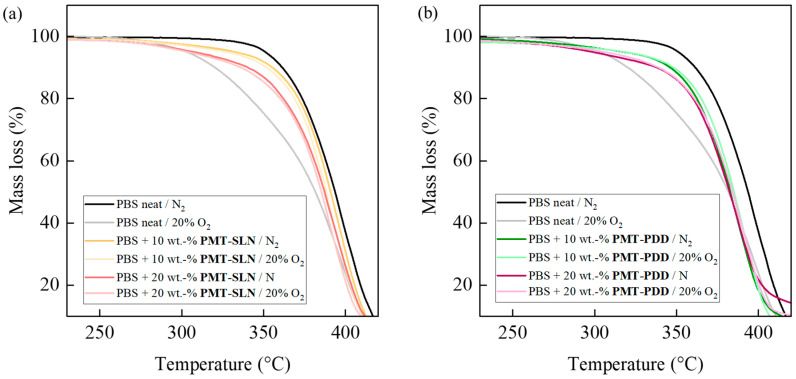
Thermal and thermo-oxidative stability of neat BioPBS and PMT biocomposites: TGA mass loss curves measured in a nitrogen and oxygen atmosphere for (**a**) PMT-SLN and (**b**) PMT-PDD biocomposites.

Figure 14 shows the TGA mass loss curves of BioPBS neat and PMT composites in 100% N_2_ and a N_2_/O_2_ mixture. As expected, the neat BioPBS shows significantly earlier thermo-oxidative decomposition under normal atmospheric conditions (20% O_2_). The BioPBS decomposition is supposed to start with α-hydrogen abstractions and subsequent building of polymerized hydroperoxide intermediates. Dependence on the thermo-oxidative stress and present molecules different oligomers causes various end-groups to form. The addition of both PMT fillers can prevent radical chain reactions due to the deactivation of the free radicals and thus stabilize the BioPBS composite from decomposition. PMT-SLN performs particularly well, matching the mass loss curve in 100% nitrogen atmosphere at 10 wt.-%. The onset temperature is about 20 °C higher than for the unstabilized neat BioPBS (Table 3). The composite filled with 20 wt.-% PMT-SLN is still stabilized against thermo-oxidative decomposition, but to a lesser extent. The stabilization effect of PMT-PDD is less pronounced and also shows no difference between 10 wt.-% and 20 wt.-%. A further stabilization against thermal degradation could not be detected, even though the PMT-SLN shows a higher thermal stability compared to the PMT-PDD. The PMT-PDD showed higher concentrations of lignin with no sufficient influence on the thermal degradation.

The higher stabilizing effect of PMT-SLN does not coincide with the results of TPC and AA. According to these analyses, PMT-PDD had both a higher concentration of total polyphenols and a higher antioxidant activity. Benítez et al. [43] investigated PMT as dietary fibers and found out, that some polyphenols might just act antioxidative after extraction from the fibers at higher temperatures. They found higher TPC and ABTS for parchments after an extrusion at 160 °C compared to 135 °C extrusion. The majority of polyphenols in PMT were found to be soluble with varying concentrations in dependence of variety and processing [37]. Thus, it was concluded that the insoluble number of polyphenols that might be bonded covalently on the cell walls of the dietary fibers can be extracted just at higher energy levels like in the extrusion at higher temperatures.

This is comparable to the roasting of coffee, where aromas and acids are to be produced by means of roasting time and temperature. Opitz et al. [49] showed that an optimum of AA can be reached at lower roasting temperatures around 190 °C, which is in good accordance to the melting temperatures during compounding. Higher roasting temperatures lead to a reduced AA and higher mass loss. It might be concluded that the polyphenols are released parallel to the BioPBS macromolecular degradation and act as spontaneous radical scavengers. The higher dietary fiber and homogeneous PMT content of the PMT-SLN seem to be advantageous compared to PMT with COC residues. Since soluble polyphenols themselves may also be subject to degradation during extrusion, the dietary fibers thus provide a kind of protective carrier for the antioxidants that are effective and efficient for the BioPBS. Table 4 summarizes the main TGA parameters under oxygen atmosphere.

**Figure 15:**
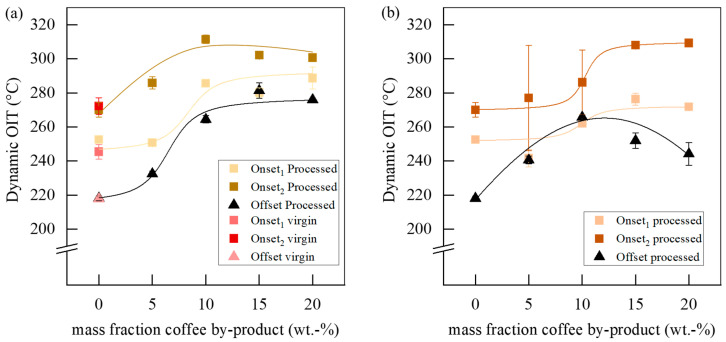
Influence of mass fraction of (**a**) PMT-SLN and (**b**) of PMT-PDD on the Dynamic OIT of the BioPBS composite.

The same stabilization effects and differences between the PMT-SLN and PMT-PDD composites could be confirmed with dynamic OIT measurements (Figure 15). With the offset method first radical reactions can be indicated. The onset OIT temperatures of the progressive oxidation were determined tangentially from the maxima of the first deviation. As described above, thermo-oxidative, degradation can occur in multiple steps, as also reported by Rizzarelli et al. [24]. In addition to the α-hydrogen abstraction mechanisms and the formation of unstable hydroperoxide intermediates, other oxidation reactions could lead to the formation of macroradicals, which are favored by higher temperatures. The released antioxidants from the PMT fibers reduce the free radicals by forming their own less stable radicals, prevent the formation of macroradicals or eliminate chain reaction triggers by radical scavengers. Higher onset and offset temperatures compared to the virgin BioPBS could already be seen at 5 wt.-% for the PMT-SLN, reaching a maximum at 10 wt.-% of the by-product. For the PMT-PDD composites, a first stabilizing effect was just found for the offset temperature at 5 wt.-% with the same maximum at 10 wt.-% PMT-PDD. The effect on the onset temperatures could be detected earliest at 10 wt.-% with a maximum at 15 wt.-%.

Wheres the PMT-SLN composites showed a more steady state plateau after the maximum OIT according to the offset method, the PMT-PDD composite offset OIT decreased with a higher concentration of the PMT-PDD. For the onset method, an inverse relationship is shown. Here, the second onset of the PMT-SLN also shows a decrease of the OIT after reaching the maximum at 10 wt.-%, while the PMT-PDD reaches a stationary pleatau. A reason for maximum stabilizing concentrations might be the pro-oxidative effect, reported in [50,51,52]. Excessive concentrations of antioxidants can lead to adverse molecular reactions, either by H-abstraction or the formation of macroradicals, which lead to further cleavage products and support a degradation of BioPBS macromolecules.

It could be shown that PMT has the potential to stabilize biopolyesters against thermo-oxidative degradation, but not against thermal degradation. The homogeneity of the parchments appears to be advantageous here, since coffee cherry residues and the sugars they contain can lead to a stronger brown discoloration and less effective stabilizing effect against thermal-oxidative decomposition. Further investigations should consider the potential Maillard reactions and their contribution to disadvantageous side reactions. The decisive factor is the dissolution of initially insoluble polyphenols from the fibers at higher temperatures, which until then are themselves protected from metabolic processes in the fibers. Therefore, homogeneous PMTs are very suitable as natural stabilizers for BioPBS when properly prepared.

## 4. Conclusions

In this study, PMT was used to investigate an approach to stabilize BioPBS that allows conventional petrochemical stabilizers to be eliminated and fully bio-based composites to be produced. PMTs are available in coffee producing countries as cheap by-products. Their utilization can reduce the overall costs of still expensive biopolymer composites and increase their stability against thermo-oxidative decomposition. For the incorporation of the PMTs into the biopolyesters matrix, they first had to be micronized into a fine powder with an extraction of the free moisture. The milldrying using a TurboRotor^®^ was proved to be a very effective method for micronizing thermally sensitive materials in order to obtain a dry lignocellulosic powder with a large proportion of antioxidants. The polyphenol content, radical scavaging properties and antioxidative activity were analyzed by spectrophotometric assays, resulting in moderate values. Their true stabilizing potential could only be demonstrated by OIT and TGA analyses after extrusion into BioPBS composites. The insoluble polyphenols contained in the PMT fibers appear to be primarily responsible for the thermal-oxidative stabilization, which are only released at higher temperatures in the extrusion, precisely when free radicals are formed in the BioPBS. A maximum of stabilizing effects could be found at 10 wt.-%. A higher concentration of antioxidants can even negativly impact the stabilization. Homogeneous PMT from fully washed coffee processing without residues of other coffee shells like coffee cherries showed a better real stabilizing effect. A higher content of cellulose in PMT leads to higher tensile moduli. Higher fiber length and diameter can negativly effect the elongation at break and need to be prevented. PMT could be succesfully utilized as cheap, free-available fully biogenic thermo-oxidative stabilizers for biobased BioPBS.

## Figures and Tables

**Table 1:** Properties of the native and milldried coffee parchments (DG = dry-grinded or milldryed, *a_w_* = water activity, D_97_ = top cut, TPC = total polyphenol content, AA = antioxidative activity).

	H_2_O (%)		*a_w_* (-)		D_97_	TPC	AA_DPPH_	AA_ABTS_
	native *	DG **	native	DG	µm	mg GAE/g dw	TEAC (mmol TE/100 g dw)	
PMT-PDD	10.3	1.79	0.47	0.14	93.5	8.46	2.75	5.36
PMT-SLN	7.0	1.16	0.32	0.12	168.2	7.47	0.77	2.94

* equilibrium moisture content at 23 °C, 50% r.H./** direct after milldrying.

**Table 2:** TGA data of both milldried coffee parchments in the temperature range from 50–550 °C with the determination of characteristic compounds (T_10%_ = temperature at 10% mass loss; T_a_ onset temperature; DTG: peak temperature derivative TG curve; Δm = mass loss of the lignocellulosic fractions; W = water; HC = hemicelulose; C = cellulose; L = lignin).

	T_a_	T_10%_	DTG	Δm_W_	Δm_HC_	Δm_HC_	Δm_C_	Δm_L_	m_residual_
	°C	°C	°C	%	%	%	%	%	%
PMT-SLN	302.5	285.5	364.4	1.0	0.5	15.4	54.4	6.79	20.9
PMT-PDD	272.7	242.6	329.6	1.6	6.7	10.4	40.2	13.9	26.3

**Table 3:** Thermal properties of the PMT-composites.

	PMT-SLN Composites						PMT-PDD Composites					
Mass Fraction	T_g_	T_m_	X_c_	T_10%_	T_on_	DTG	T_g_	T_m_	X_c_	T_10%_	T_on_	DTG
wt.-%	°C	°C	%	°C	°C	°C	°C	°C	%	°C	°C	°C
0	−25.0	116.5	34	361.7	367.5	398.8	−25.0	116.5	34	361.7	367.5	398.8
5	−27.4	117.0	33	-	-	-	−23.8	118.4	35	-	-	-
10	−26.9	116.5	35	356.5	367.5	396.1	−31.3	117.7	36	351.7	362.8	390.9
15	−27.6	117.3	34	-	-	-	−27.5	117.1	38	-	-	-
20	−28.5	116.1	34	343.7	360.9	394.4	−27.8	116.6	36	341.5	358.7	391.5

**Table 4:** TGA data for the thermo-oxidative Properties of PMT-composites.

	PMT-SLN			PMT-PDD		
Filler Content	Ox. T_10%_	Ox. T_on_	Ox. DTG	Ox. T_10%_	Ox. T_on_	Ox. DTG
wt.-%	°C	°C	°C	°C	°C	°C
0	320.4	347.9	397.1	320.4	347.9	397.1
10	353.2	365.2	392.6	347.0	358.3	385.9
20	339.1	360.6	392.8	340.1	355.4	386.6

## Data Availability

The data presented in this study are available on request.
